# A location semantic privacy protection model based on spatial influence

**DOI:** 10.1038/s41598-025-88553-9

**Published:** 2025-04-30

**Authors:** Linghong Kuang, Wenlong Shi, Xueqi Chen, Jing Zhang, Huaxiong Liao

**Affiliations:** 1 School of Computer Science and Mathematics, Fujian University of Technology, Fuzhou, 350118 China; 2 Fujian Provincial Key Laboratory of Big Data Mining and Applications, Fuzhou, 350118 China; 3 Faculty of Data Science, City University of Macau, Macau, 999078 China

**Keywords:** Semantic privacy, *K*-anonymity, Diversified semantic, Spatial influence, Dummy trajectory synthesis, Computer science, Information technology

## Abstract

The utilization of numerous location-based intelligent services yields massive traffic trajectory data. Mining such data unveils internal and external user features, offering significant application value across various domains. Nonetheless, while trajectory data mining enhances user convenience, it also exposes their privacy to potential breaches. To address the problem that existing traffic trajectory privacy protection methods rarely consider the location semantics and the spatial influence of interest points when constructing *k*-anonymity sets, which makes user trajectories vulnerable to attacks, a Location Semantic Privacy Protection Model based on Spatial Influence (LSPPM-SI) is proposed to resist semantic attacks. Firstly, a location semantic mining algorithm is proposed to classify the stopovers based on positional semantics, thereby simplifying the semantic information contained in user trajectories. Secondly, a diversified semantic dummy location selecting algorithm is proposed to resist semantic attacks. To enhance the availability of traffic trajectory data while safeguarding location semantics, a Hilbert curves is used to reduce the area of anonymous regions, and a diversified semantic anonymous set is constructed. Thirdly, the spatial influence of interest points is defined and used to verify the rationality of dummy trajectories within the anonymous trajectory set, thereby preventing attackers from identifying dummy trajectories. Finally, the problem of synthesizing dummy trajectories is transformed into a matching problem for directed bipartite graphs and the optimal *k*-anonymity set is obtained using the Kuhn Munkres algorithm. Experimental results demonstrate that the proposed model improves traffic trajectory data availability and semantic protection performance by 14% and 46.5%, respectively, compared to traditional models.

## Introduction

With the advent of 5G wireless technology, mobile intelligent terminals have experienced significant development. Users now have the ability to query location-related service information at any time, enjoying convenient and rapid services^1,2^. However, users also face the risk of privacy leakage while utilizing location-based services, as service providers store users’ traffic trajectory data^3,4^. These data encompass a wealth of sensitive information, including mobile user shopping habits, property or home address, workplace details, and frequently visited places. If the service provider is untrustworthy or maliciously exploited, trajectory data may be directly released without protection, posing a serious threat to mobile users’ sensitive information and personal privacy and security^5^.

With the growing emphasis on privacy and personal security, unresolved issues regarding traffic trajectory privacy leakage may soon impede the advancement of location-based intelligent services. Currently, researchers have employed various location privacy protection methods to mitigate privacy leakage, many of which rely on *k*-anonymity technology^6–8^. This technology constructs *k*-anonymity sets to render mobile users’ trajectories indistinguishable from other $k-1$ trajectories. However, effective construction of *k*-anonymity sets poses significant challenges. Attackers can leverage background information to access all published traffic trajectory data and identify dummy trajectories within anonymous sets through data mining techniques^9^. To address some of the limitations of *k*-anonymity, *l*-diversity was introduced as an enhancement. This approach ensures that each equivalence class in the anonymized dataset contains at least *l *distinct sensitive values, which helps mitigate homogeneity and background knowledge attacks by increasing attribute diversity^10,11^. However, while *l*-diversity can effectively defend against certain types of inference attacks, it does not address the specific challenges posed by semantic attacks or complex inference that considers location semantics and spatial relationships. To address these challenges, various methods have been developed to safeguard user traffic trajectory privacy, with a focus on mitigating the risks associated with semantic attacks. For instance, numerous existing methods utilize similarity measures to filter trajectories^12,13^. These methods also assess the coherence of adjacent anonymous sets by considering factors such as time reachability and directional similarity in entry and exit degrees. Despite these efforts, existing approaches still fall short in effectively countering semantic attacks, highlighting the need for more comprehensive privacy protection techniques.

**Fig. 1:**
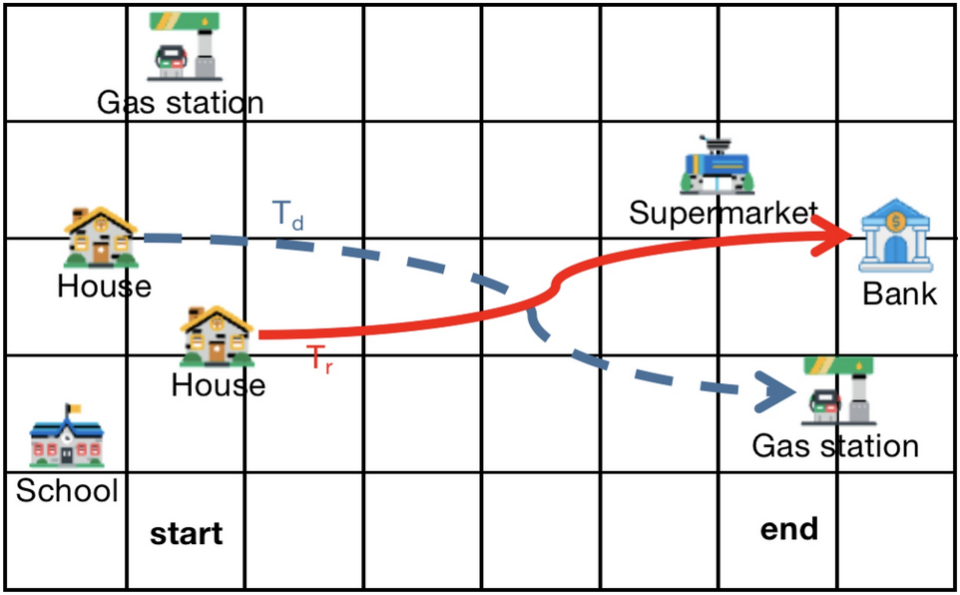
Anonymous results without resisting semantic attacks.

The limitations of models neglecting semantic attacks are illustrated in Figure 1. For example, a method that overlooks semantic attacks may produce an anonymous set with $k=2$. In the figure, the red solid line represents the user’s actual traffic trajectory, indicating a journey from home to a bank. Notably, there are residential areas, a school, and a gas station near the trip’s origin, while a supermarket and another gas station are located near the trip’s end. The blue dashed line depicts the generated dummy trajectory. This method exhibits two main drawbacks: (1) Both the real and dummy trajectories originate from residential areas, allowing attackers to infer that the user’s home lies within a small vicinity of the starting point. (2) The presence of a gas station near the anonymous area implies that users would likely opt for the nearest refueling station. This inclination increases the likelihood of attackers identifying such trajectories as dummy trajectories with higher probability. Consequently, anonymous sets generated by methods lacking defenses against semantic attacks pose a risk of privacy leakage.

Moreover, while semantic-based anonymity methods attempt to address some of these issues by incorporating positional semantics into privacy protection, they still face limitations in real-world applications. Semantic methods often lack flexibility when protecting dynamic, high-frequency trajectory data because they focus on predefined semantic categories, which may not cover all possible user locations. Additionally, these methods may struggle with balancing privacy protection and data availability; efforts to preserve semantic meaning often expand the anonymous area, reducing the data utility and complicating real-time applications. Semantic homogeneity within anonymous sets is another challenge, as grouping locations with similar semantics can make it easier for attackers to deduce real positions.

It’s evident that existing *k*-anonymity models, including dummy and semantic-based methods, still face several challenges in safeguarding the privacy of traffic trajectory location: (1) Ensuring the protection of semantic attributes of locations from attacks while safeguarding user location information. (2) Developing methods to construct anonymous sets that minimize the anonymous area, thereby enhancing data availability while protecting location semantics. (3) Enhancing the resemblance between dummy trajectories and real traffic trajectory data to the extent that attackers cannot differentiate between them.

**Fig. 2:**
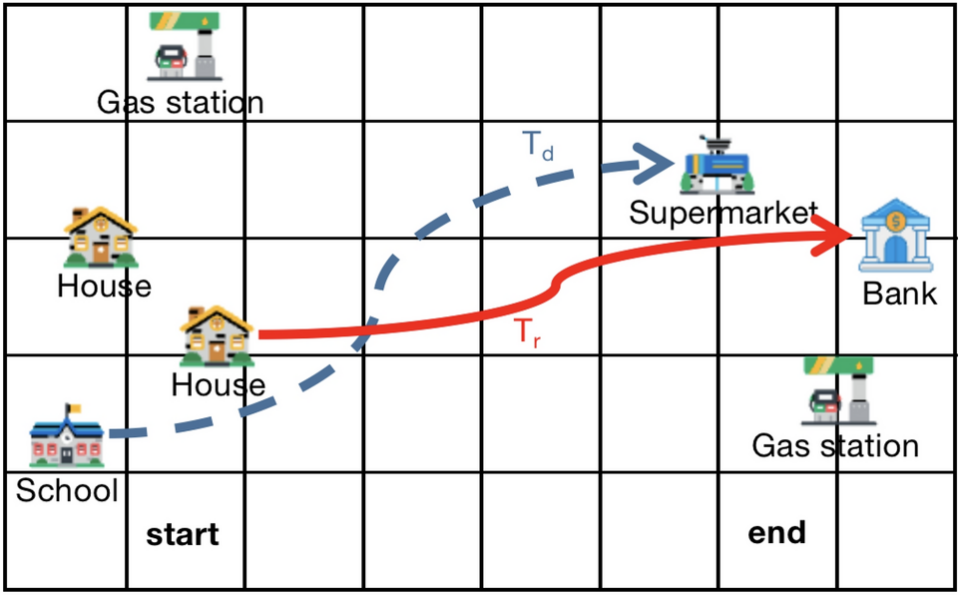
Anonymous results with resisting semantic attacks.

In other to address these challenges, a Location Semantic Privacy Protection Model based on Spatial Influence (LSPPM-SI) is proposed in this paper. The motivation of LSPPM-SI is shown intuitively in Figure 2. As illustrated, unlike the user’s real trajectory, the dummy trajectory begins at the school. Furthermore, there is only one supermarket depicted on the map. When a user intends to visit a supermarket, it’s highly probable that they would choose this specific one. This aspect makes it difficult for attackers to confidently discern the dummy trajectory (represented by the blue dashed line) from the real trajectory, thereby safeguarding the user’s true trajectory.

Specifically, stopovers within trajectories are first extracted, and a location semantic mining algorithm is introduced to determine the semantics associated with different stopovers. Secondly, the Hilbert curve is used to reduce the area of anonymous regions, thereby enhancing the availability of traffic trajectory data. Concurrently, a diversified semantic dummy location selection algorithm is introduced to thwart semantic attacks. Thirdly, the spatial influence of points of interest is defined and used to assess the rationality of dummy positional connections between adjacent anonymous sets, aiming to prevent attackers from identifying dummy trajectories. Finally, the problem of generating dummy trajectories is transformed into a matching problem for directed bipartite graphs, and the optimal anonymous set of synthetic trajectories is obtained using the Kuhn Munkres algorithm. The main contributions of this model are summarized as follows:

1. In order to achieve semantic protection for user’s stopovers, a location semantic mining algorithm is proposed. This algorithm partitions different stopovers and simplifies the representation of user trajectories, thereby reducing the computational complexity of the algorithm.
2. In order to reduce the area of anonymous regions and enhance data availability, the Hilbert curve is used to select candidate dummy positions that are closer to the real positions.
3. In order to resist location semantic inference attacks, a diversified semantic dummy location selecting algorithm is proposed. This algorithm safeguards the user’s location semantics by ensuring that the anonymous set contains a broader range of semantic categories.
4. In order to synthetize dummy trajectories that are challenging for attackers to discern, the concept of spatial influence factor is proposed to filter out obvious dummy trajectories. Subsequently, the task of synthesizing dummy trajectories that closely resemble real trajectories is transformed into a directed bipartite graph matching problem. The Kuhn Munkres algorithm is used to derive the optimal anonymous set of dummy trajectories.
5. This article proposes a location privacy protection model based on spatial influence. Theoretical analysis and experimental results demonstrate that LSPPM-SI can better protect user’s location semantic privacy while ensuring the availability of traffic data.

The rest of the paper is organized as follows: In Related Work section, existing researches related to our work are reviewed.Preliminaries section introduces some theories and basic definitions. Methodology section introduces the location semantic privacy protection model proposed in this paper. In section Experiments, experimental evaluations are conducted to evaluate the effectiveness of LSPPM-SI. Finally, the conclusions and future works are presented in section Conclusions.

## Related work

The primary objective of protecting traffic trajectory privacy is to prevent attackers from accessing sensitive user information while maintaining data availability. Various technologies have been proposed for this purpose, including data desensitization, anonymity, encryption, and perturbation technologies. Data desensitization technology aims to protect user privacy by obstructing or obscuring sensitive information^14^. However, attackers can often deduce sensitive user information through background knowledge despite these measures. Encryption technology encrypts the user’s location information, making it completely invisible to the server^15^. This approach can meet stringent privacy protection requirements while ensuring service availability. However, encryption technology often necessitates additional hardware support, leading to significant computational and communication overhead. Consequently, it may not be suitable for encrypting large volumes of traffic data. Perturbation technology safeguards user privacy by introducing suitable noise into published data, without taking into account the attacker’s background knowledge^16^. However, these methods are primarily applicable to protecting digital data. Additionally, when user data is interrelated, perturbation significantly diminishes the utility of protected data and poses a potential risk of leakage. *k*-anonymity-based technology offers an effective solution to protecting correlated trajectory data. Introduced by Gruteser *et al.*^17^ in 2003, it ensures that each record is indistinguishable from at least $k-1$ other records, thereby reducing the probability of attackers identifying a specific user to less than 1/*k*. In this section, Three traffic trajectory privacy protection methods based on *k*-anonymity technology related to the proposed method in this paper will be introduced as follows.

### Obfuscation and dummy location methods

Obfuscation and dummy location methods aim to mask or generalize trajectory points to achieve *k*-anonymity in privacy protection. The obfuscation method generalizes all sampling points on a trajectory to their corresponding anonymous regions, thereby constructing *k*-anonymity sets and ensuring that each sampled point falls within an obfuscated region^18^. Albouq *et al.*^19^ propose a Double Obfuscation Approach (DOA), which continuously applies two stages of obfuscation, enhancing privacy for users in dynamic settings. Similarly, Qiu *et al.*^20^ develop a position confusion strategy that leverages geographical indistinguishability in road networks, while Bostanipour *et al.*^21^ utilize semantic label obfuscation to further prevent inference attacks.

The dummy location method, on the other hand, constructs anonymous sets by generating dummy trajectories with similar dummy positions, masking actual data points while preserving statistical properties^22^. Xu *et al.*^23^ maximize anonymity entropy for dummy selection, and Shaham *et al.*^24^ introduce temporal and directional constraints for realistic dummy paths. Zhang *et al.*^25^ further integrate semantic differentiation to enhance privacy.

### LKC-Privacy method

An extension of *k*-anonymity, the LKC-Privacy model offers a more flexible framework by guaranteeing that each published record is indistinguishable from at least *k* other records, while sensitive attribute combinations appear at least *c* times within any given *L*-dimensional context. Originally designed for RFID data protection, LKC-Privacy has proven adaptable for various data types, including trajectory data, due to its robust anonymization capabilities^26^. Riyana *et al.*^27^ assess its adaptability in high-dimensional and missing-value contexts. Subsequently, Riyana *et al.*^28^ introduce improvements for privacy preservation in RFID data-collections, demonstrating higher security and efficiency compared to LKC-Privacy. Later, Riyana *et al.*^29^ investigate anonymization in high-dimensional data through local and global data suppressions. Finally, Riyana *et al.*^30^ explore privacy-enhancing data aggregation methods for big data analytics, contributing to enhancing the utility of LKC-Privacy in handling sophisticated data aggregation scenarios. However, balancing semantic information preservation remains a challenge, especially in trajectory datasets where location semantics play a critical role. This paper builds on these insights by incorporating semantic-based techniques into the LKC-Privacy framework, aiming to enhance its applicability for trajectory data while providing more comprehensive protection against sophisticated inference attacks.

### Semantic anonymity-based method

The semantic anonymity-based method involves incorporating positional semantics to counter positional homogeneity attacks and positional semantic attacks during the construction of *k*-anonymity sets. This approach aims to make it challenging to differentiate synthetic trajectories from real trajectories, thereby safeguarding user trajectory privacy^31^.

Tan *et al.*^32^ propose a semantic trajectory anonymization model based on *k*-anonymity. This model creates a sensitive area containing $k-1$ interest points similar to the sensitive points based on the user’s movement patterns, such as walking, running, and driving. Qiu *et al.*^33^ propose a Mobile Semantic Perceived Privacy Model (MSP), which initially characterizes a new type of user-related mobile semantic location set by constructing a hierarchical semantic tree based on the user’s role in the location. Subsequently, a dedicated method is introduced to assess the privacy sensitivity of the location and integrate it into user-related mobile semantics. Finally, an adaptive privacy protection mechanism MSP is developed, fully considering the personalized needs of users and locations. Zheng *et al.*^34^ present a novel semantic-aware privacy protection online location trajectory sharing mechanism to safeguard data privacy and semantic privacy while maintaining the utility of semantic-aware data. They propose two new privacy metrics to quantify data privacy leakage and semantic privacy leakage, respectively. Additionally, they introduce a semantic-aware utility metric to quantify the utility of semantic-aware trajectory data. By formulating a multi-objective optimization problem, they address the limitation of ensuring data utility in the privacy protection process. Qiu *et al.*^35^ present a posterior behavioral-semantic privacy-preserving solution (BSPri), which addresses privacy risks associated with published traces by simulating inference attacks. By generating synthetic trajectories with similar anonymity semantics, BSPri makes it challenging for attackers to infer dummy trajectories, thus safeguarding the user’s positional semantic information. Li *et al.*^36^propose a Semantic-based Location Privacy Protection (SLPP) algorithm that leverages knowledge graphs to enhance location semantic security. This approach considers temporal semantics, positional semantic sensitivity, and physical spatial distance to generate an anonymous candidate set. Subsequently, by evaluating the semantic similarity of positions, the anonymous candidate set is refined and discretized between positions to derive the final secure anonymous set.

GAN-based methods are increasingly used for user trajectory privacy protection, with techniques that produce realistic synthetic trajectories while preserving statistical and spatiotemporal properties. Rao *et al.*^37^ leverage LSTM networks to capture temporal dependencies, and Ma *et al.*^38^ extend this with Transformer-based models for semantic encoding. Although GAN-based methods provide high-quality dummy trajectories, they are computationally intensive and can still be vulnerable to context-based attacks.

Each of these *k*-anonymity-based methods offers unique advantages and drawbacks. Obfuscation methods maintain data authenticity but have high computational costs, and dummy location methods are easy to implement yet may create storage burdens due to large volumes of dummy data. LKC-Privacy provides a flexible anonymization framework that can be adapted for trajectory data but faces challenges in balancing privacy with semantic preservation. Semantic anonymity methods protect location semantics but can be costly and might not address semantic homogeneity attacks comprehensively. To address these limitations, this paper proposes a novel model that synthesizes anonymous sets comprising semantically similar trajectories, making it challenging for attackers to distinguish real data while maintaining data availability and protecting user privacy effectively.

## Preliminaries

In this section, several definitions related to traffic trajectory data are first introduced. Subsequently, two attack models will be presented along with specific examples for clarification. Finally, the dummy trajectory privacy protection method based on *k*-anonymity and problem formalization will be introduced.

### Definition of traffic trajectory data

The following provides formal definitions related to traffic trajectories.

#### Definition 1

*Trajectory*. This paper assumes that time and space are discrete. Therefore, the user’s traffic trajectory *T* is considered as a three-dimensional line, comprising a sequence of sampling points accessed over time. The traffic trajectory *T* is represented as $T=\left\{ p_1,p_2,...,p_i,...,p_n \right\}$. Each sampling point $p_i (i=1,2,...,n)$ of the trajectory is denoted by a triplet $<x_i,y_i,t_i>$, where $<x_i,y_i>$ represents the coordinates of the sampling point, and $t_i$ is the sampling timestamp. The traffic trajectory is generated by the user’s daily travel, which may involve various modes of transportation such as walking, cycling, driving cars, taking trains, airplanes, etc. There are two types of sampling points for traffic trajectories: passing points and stopping points. Passing points denote the positioning points where users pass through at relatively high speeds, typically during transportation journeys. Stopping points, on the other hand, refer to positioning points where a user remains stationary for an extended period in a small spatial area, with a speed of approximately 0*m*/*s* or much lower than the normal speed.

#### Definition 2

*Stopover*. The stopover of the user’s traffic trajectory is represented as $S=\left\{ s_1,s_2,...,s_j,...,s_n \right\}$, where the sub-stopover $s_j$ is a trajectory segment composed of a continuous set of stopping points, represented as $s_j=\left\{ p_1,p_2,...,p_n \right\}$. When users pass through a spatial area and stay for a sufficient amount of time, this paper considers that the user has either visited the area or performed an activity. Therefore, the user’s stopover can also be represented by a quadruple $<x,y,t_{in},t_{out}>$, where $<x,y>$ denotes the cluster center of the stopover, $t_{in}$ and $t_{out}$ represent the arrival time and departure time of the stopover, respectively. A stopover describes a specific location or place that holds significance for the user, such as a bus stop, market, or even the user’s residence. These places reveal the semantic purpose of user movement trajectories. Consequently, it is possible to mine user stopovers to analyze the movement semantics of user trajectories.

**Fig. 3:**
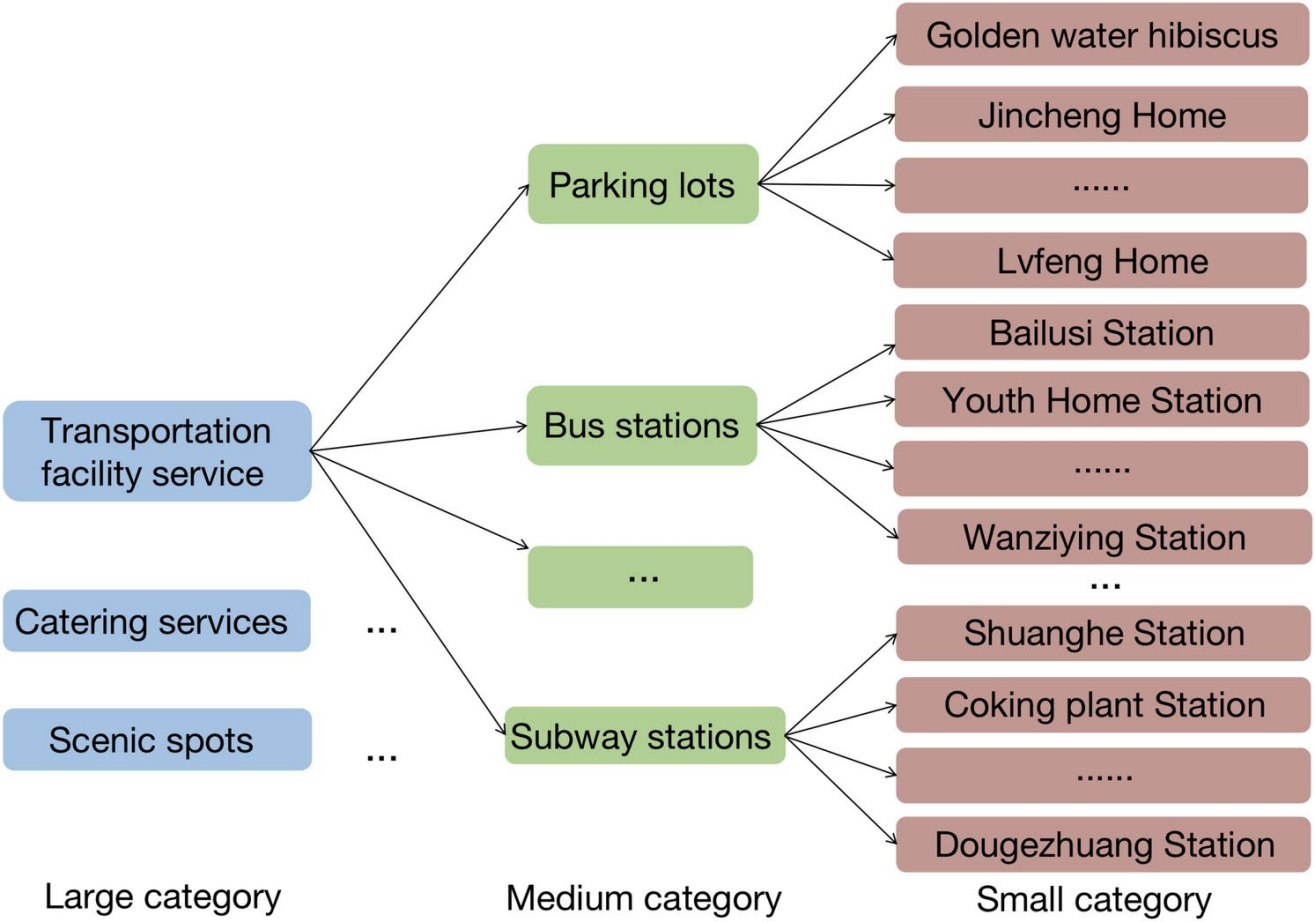
Multi-class nested positional semantic set.

#### Definition 3

*Location Semantics*. Location semantics refer to the semantic attributes associated with traffic trajectory sampling points, which represent the functions or services provided by the user’s location. Real-world location semantics encompass various categories, such as apartments, shopping malls, clubs, office buildings, etc. As shown in Figure 3, this paper uses multi-class nested location semantic sets to depict different semantic category levels, which are categorized into three levels: large category, medium category, and small category. For example, the large category includes transportation facility services, catering services, scenic spots, etc. Under the transportation facility service category, there are subcategories such as parking lots, bus stations, subway stations, etc. The subway station subcategory further includes specific stations like Shuanghe Station, Coking Plant Station, Dougezhuang Station, etc., representing various levels of granularity within categories. Locations belonging to the same large category may possess different semantic attributes, such as business hours and user access time, whereas those within the same medium category typically exhibit similar semantic attributes. Additionally, different semantic positions carry distinct semantic sensitivities.

#### Definition 4

**Fig. 4:**
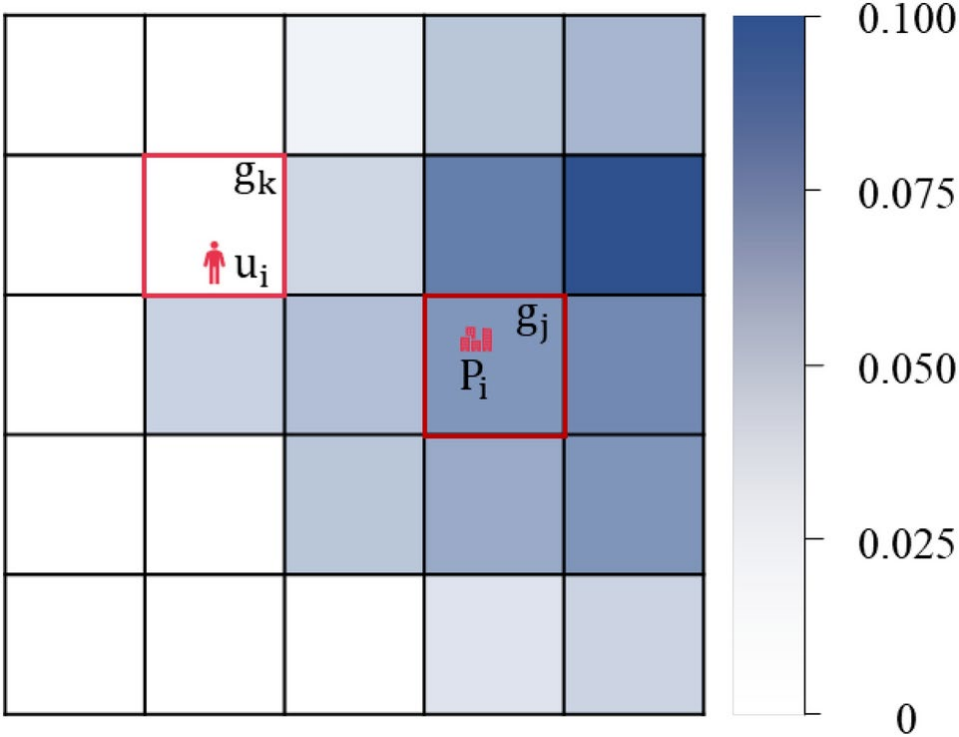
Spatial influence distribution map. The darker the color, the greater the spatial influence of interest points within the grid.

*Spatial Influence*. As shown in Figure 4, the point of interest $P_i$ is located in grid $g_j$, while user $u_i$ is positioned in grid $g_k$. Notably, grid $g_j$ may potentially coincide with $g_k$. By partitioning the geographic space into grids and assuming user transitions between grids according to Markov models, the spatial influence of the point of interest $P_i$ on grid $g_k$ is computed using Equations (1).

1 $$\begin{aligned} I_i=Pr(g_j|g_k)\cdot Pr(P_i|g_j) \end{aligned}$$

where $Pr(g_j|g_k)$ denotes the transition probability from grid $g_k$ to $g_j$, which can be derived from real user mobile datasets and trained using gravity models^39^. $Pr(P_i|g_j)$ represents the frequency with which user $u_i$ accesses $P_i$ in grid $g_j$, and it can be obtained through kernel density estimation to achieve a smooth distribution.

In this paper, the attributes of interest points are represented by spatial influence. Spatial influence quantifies the extent to which a point of interest affects users in different locations. This implies that for traffic trajectories with multiple stopovers, interest points corresponding to subsequent stopovers can attract users from previous stopovers to move towards them.

### Attack models

#### Definition 5

**Fig. 5:**
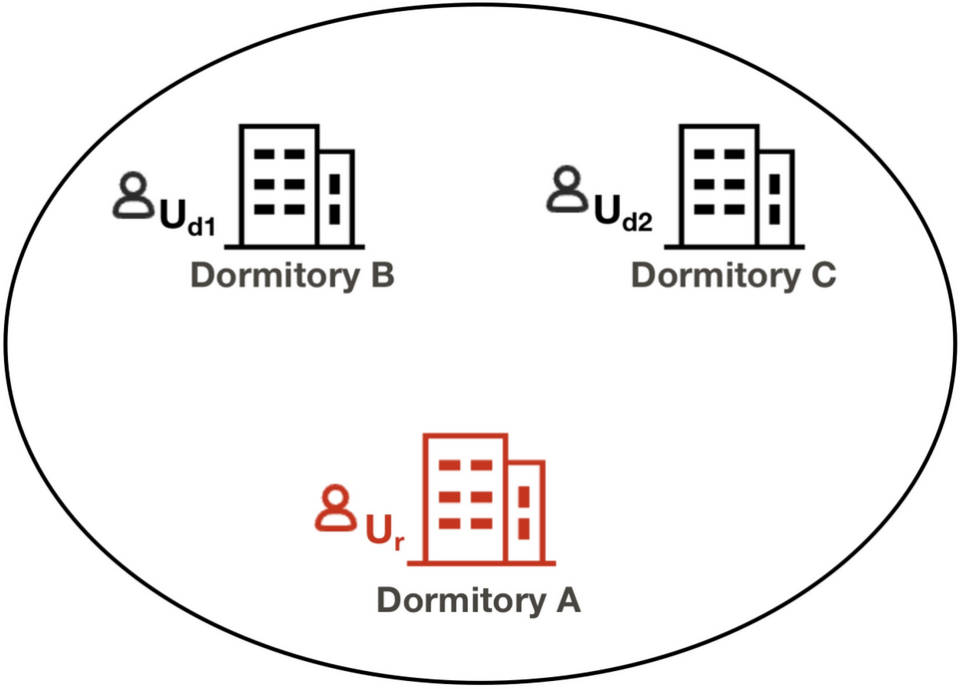
Schematic diagram of positional homogeneity attack.

*Positional Homogeneity Attack*. Positional homogeneity attack refers to the generation of an anonymous set containing *k* location distributions, where all locations are clustered within a small geographical area or share the same location semantics. This can result in any location within the anonymous set being perceived as the user’s actual location, thereby compromising their privacy. As shown in Figure 5, the generated 3-anonymous set consists of real user $U_r$ and two dummy locations $U_{d1}$ and $U_{d2}$ distributed within the student dormitory. Attackers can easily discern that the real user is located within the student residence and may infer additional details, such as the user being a student.

#### Definition 6

*Positional Semantic Attack*. Semantic attack refers to the ability of attackers to infer sensitive user information even when the semantics within the same anonymous region are different. The attacker in this paper aims to discern the activities performed by the user at specific locations. Leveraging interest point data, attackers can extract semantic information based on the attributes of interest points, such as their primary socio-economic activity functions. Meanwhile, the location semantics of users encompass the semantic types of users, including their residences, workplaces, entertainment venues, etc., where users typically initiate service requests. By analyzing the user’s location and discerning their travel motivations, combined with background knowledge, attackers can obtain user privacy information.

**Fig. 6:**
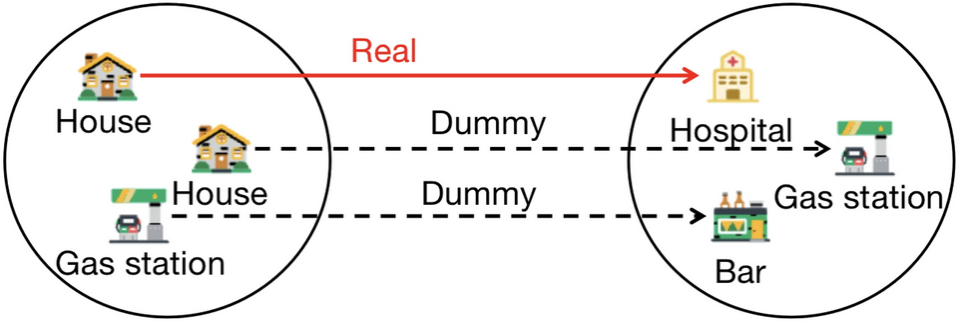
Schematic diagram of positional semantic attack.

As shown in Figure 6, there exists a generated 3-anonymous trajectory set. The real trajectory depicts movement from the house to the hospital, while the two other trajectories illustrate movement from the house to the gas station and from the gas station to the bar. Armed with background knowledge that it is 11 o’clock in the morning, the attacker can make a high-probability inference regarding the dummy trajectory from the gas station to the bar based on time, as bars typically operate during the night. From a geographical perspective, users might easily deduce that the trajectory from the house to the gas station is a dummy trajectory, given the presence of a gas station near the house. Typically, individuals opt for refueling at the nearest available location. Consequently, the attacker can infer that the user’s true trajectory is from the house to the hospital, potentially inferring that the user may be visiting due to a medical condition.

Hence, within the anonymous set of trajectories, it becomes imperative to ensure semantic diversity and employ spatial influence to weed out conspicuous dummy trajectories. This ensures that trajectories within the same anonymous set exhibit similar access characteristics to real trajectories in both spatial and temporal dimensions. Consequently, attackers encounter difficulties in distinguishing between real and dummy trajectories, thereby safeguarding sensitive user information.

**Table 1:** Anonymous trajectory data.

User	$P_1$	$P_2$	$P_3$
$U_r$	(1,2)	(3,3)	(2,7)
$U_{d1}$	(2,1)	(4,4)	(1,6)
$U_{d2}$	(1,3)	(2,5)	(4,7)

### Dummy trajectory privacy protection method

**Fig. 7:**
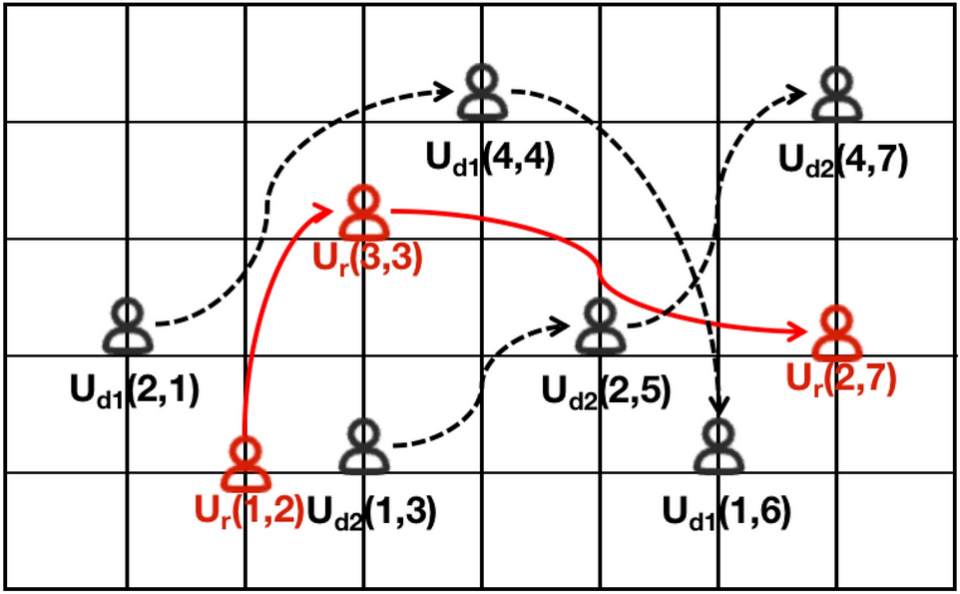
Anonymous trajectory distribution.

Dummy trajectory technology involves obscuring the user’s real trajectory among $k-1$ dummy trajectories, thereby constructing a *k*-anonymous trajectory set to shield the real trajectory and mitigate the risk of privacy leakage. The key to dummy trajectory technology is to generate $k-1$ dummy trajectories that are similar to the real trajectory, making it difficult for attackers to differentiate between them. While the implementation of dummy trajectory technology is relatively straightforward, it may lead to the generation of redundant information, and crafting dummy trajectories akin to real trajectories poses a significant challenge. For example, when $k=3$, the anonymous set of trajectories needs to construct two dummytrajectories. The trajectory data post 3-anonymization of user trajectories is presented in Table 1, while the trajectories post 3-anonymization are depicted in Figure 7. The trajectory of user $U_r$ is a real trajectory, while the trajectories of users $U_{d1}$ and $U_{d2}$ depict dummy trajectories.

**Table 2:** A summary of key notations.

Notation	Description
$p_i$	Sampling points
$\delta _{spd}$	Moving speed threshold
$\delta _{dwe}$	Dwell time threshold
$d_c$	Cut off distance based on density peak clustering algorithm
$d_r$	A radius distance threshold
$\rho _i$	Local density generated by density peak clustering algorithm
$S_i$	Stopover
$s_i$	Sub-stopover
$POI_i$	Points of interest
$\vec {poi}_i$	Feature vector of the $POI_i$
$t_{in}$	Arrival time of the stopover
$t_{out}$	Departure time of the stopover
$\delta _{sim}$	The similarity threshold of POI
$P_{ari}$	Probability of user arrival in each hour interval of the POIs
$E_{dwe}$	The expected dwell time of the POI
$V_{dwe}$	The variance of the dwell time of the POI
$g_i$	The *i*-th grid
$U_i$	User
$I_i$	Space influence
$\delta _{I}$	The spatial influence threshold
$Seq_{fsp}$	Dummy semantic position sequence
*T*	Trajectory anonymity set

### Problem formalization

The main problem studied in this paper involves the privacy risks associated with trajectory data, particularly focusing on the vulnerabilities arising from semantic attacks and spatial influence of interest points. The objective is to develop a model that effectively preserves the privacy of user trajectories by resisting semantic-based attacks while maintaining the utility of trajectory data. Specifically, the targeted goal is to construct an enhanced *k*-anonymity set that considers the semantic properties of stopovers and optimizes spatial influence factors, thus providing robust privacy protection against attackers who exploit semantic information. This involves leveraging a diversified semantic dummy location selection algorithm and utilizing the spatial influence of interest points to ensure that the dummy trajectories closely resemble real trajectories, making them indistinguishable from an attacker’s perspective. A summary of key notations used in this paper is shown in Table 2.

## Methodology

**Fig.8:**
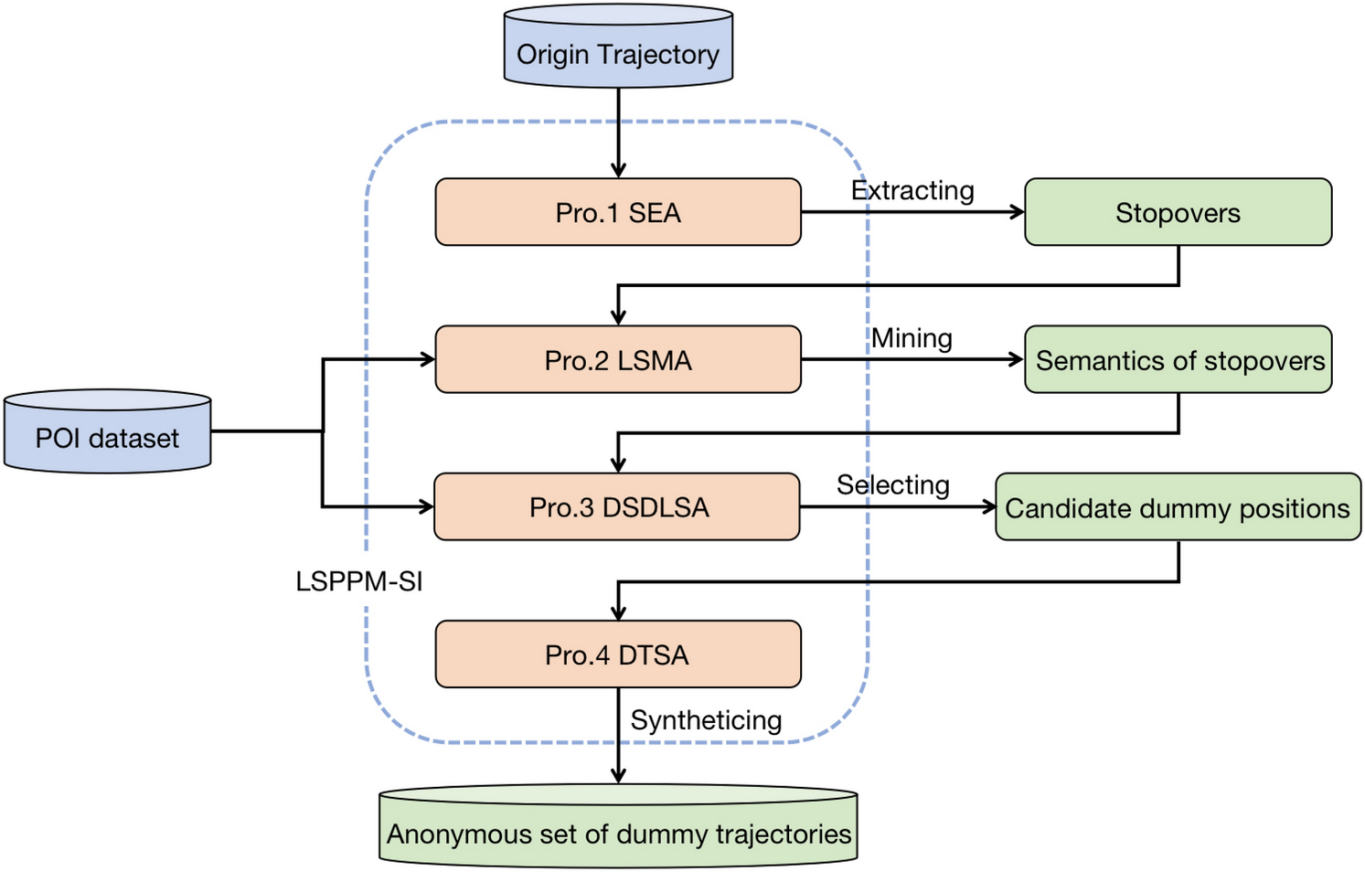
The overall framework of LSPPM-SI.

This section provides an overview of the LSPPM-SI, as shown in Figure 8. LSPPM-SI is divided into four stages: (1) Extraction of stopovers. The stopovers of a trajectory are extracted based on Stopover Extraction Algorithm (SEA) presented in Algorithm 1 to extract; (2) Mining semantics of stopovers. The position semantic attributes of the stopovers are labeled by combining the interest point dataset using the Location Semantic Mining Algorithm (LSMA) described in Algorithm 2; (3) Selection of candidate dummy positions. The positional semantics of candidate anonymity sets are diversified based on a Diversified Semantic Dummy Location Selecting Algorithm (DSDLSA) presented in Algorithm 3; (4) Dummy trajectory synthesis. The Dummy Trajectory Synthesis Algorithm (DTSA) is used to synthetic dummy trajectories based on the spatial influence of interest points in Algorithm 4.

### Extraction of stopovers

This section will introduce the stopovers extraction process in detail. A point along the user’s trajectory is considered a stopover if the user’s speed passing through that location is approximately equal to 0 m/s or much less than the normal speed. Specifically, if a user passes through a location and stays for a significant amount of time, it is inferred that the user has visited the area or performed an activity there. For instance, when the user’s speed ranges from 0 to 1 m/s, it is likely that the user is engaged in activities at the stopover. However, it’s important to note that users may utilize various modes of transportation during their travels, and these modes may encounter obstacles on the road, resulting in significantly reduced speeds. These instances of slowed movement due to road obstructions are not considered user stopovers in this study. Therefore, the moving speed threshold $\delta _{spd}$ is used to differentiate between passing points and stopping points along the traffic trajectory. A “quasi-stopover” can be formed by several stopping points. Moreover, the dwell time threshold $\delta _{dwe}$ is used in all “quasi-stopovers” to determine whether they are genuine stopovers or merely “fake-stopovers” caused by brief halts due to traffic obstructions.

User activities typically exhibit a hierarchical “multi-center” characteristic and display strong regularity^39^. Users tend to allocate most of their time to a few “major stopovers,” such as their home address and workplace, and frequently commute between these locations. Additionally, users often access some “sub-stopovers” within a limited radius of the main stopover at specific times. For example, a user might spend an extended period in the office, walking at a normal speed to a meeting room nearby, or briefly stopping at a scenic spot in the park for a few minutes before continuing to another scenic spot within the same park. Consequently, merging multiple “sub-stopovers” of users in a certain area into one “primary stopover” is considered in this paper. This approach effectively represents the user’s stay patterns while minimizing data redundancy and significantly reducing the computational cost of subsequent analysis.

**Algorithm 1:**
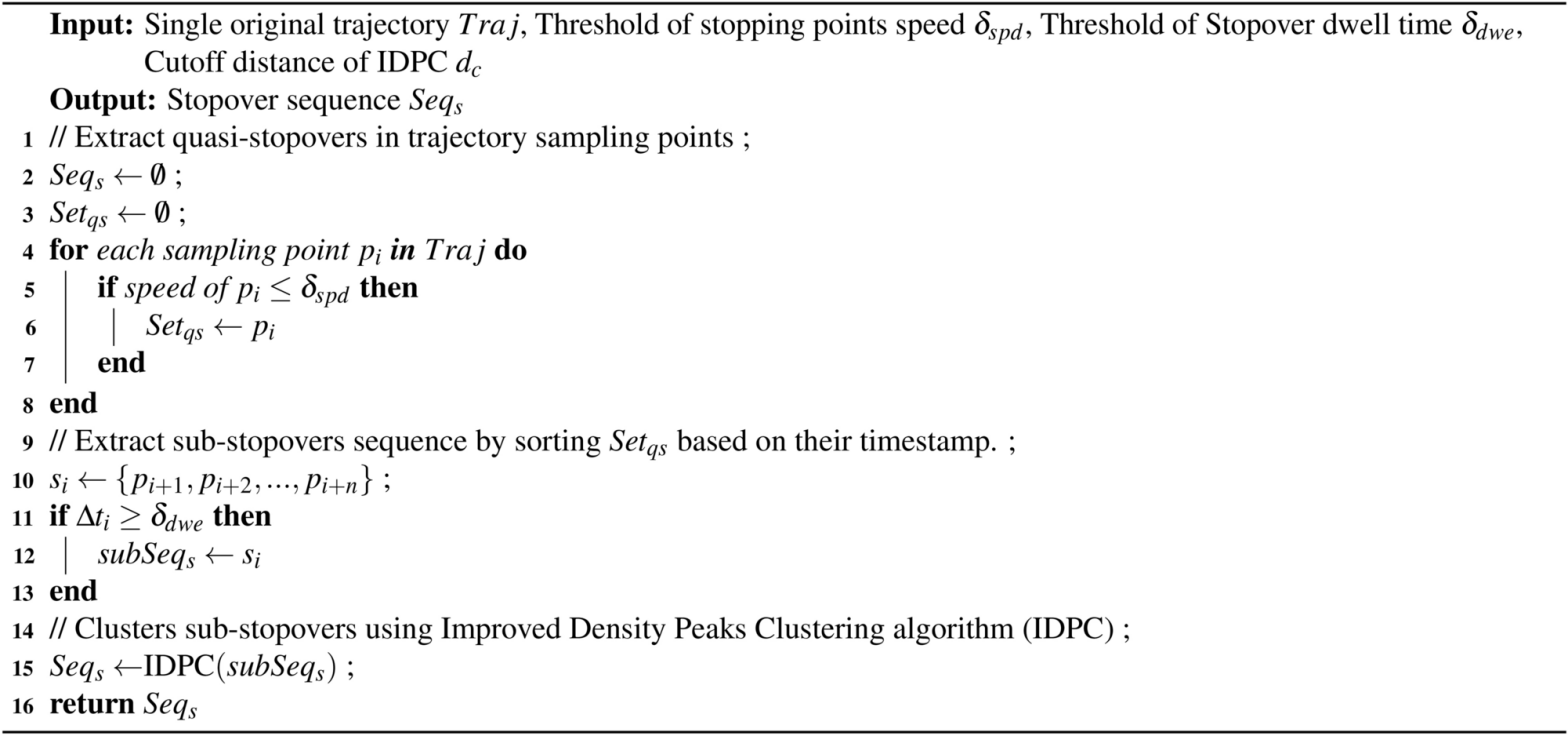
Stopover Extraction Algorithm (SEA)

Based on the above analysis, a clustering-based method for extracting stopovers is proposed in this paper. Given a sub-stopover $s=\left\{ p_1, p_2,..., p_n \right\}$ composed of a set of continuous stopping points. The coordinates of *s* are the centroid coordinates of these stopping points. The entry time of *s* is the timestamp of the first sampling point $p_1$, and the exit time of *s* is the timestamp of the last sampling point $p_n$. Therefore, *s* can be represented as an object $s=\left\langle x,y,t_{in},t_{out}\right\rangle$, and various information regarding *s* can be calculated by Equations (2).

2 $$\begin{aligned} \left\{ \begin{matrix} s.x=\frac{ {\textstyle \sum _{i=0}^{n}}p_i.x }{n} \\ s.y=\frac{ {\textstyle \sum _{i=0}^{n}}p_i.y }{n} \\ s.t_{in}=p_1.t_{in} \\ s.t_{out}=p_n.t_{out} \end{matrix}\right. \end{aligned}$$

where *s*.*x* and *s*.*y* denote the longitude and latitude of *s*, respectively, while $s.t_{in}$ and $s.t_{out}$ denote the arrival and departure times of *s*. Similarly, $p_i.x$ and $p_i.y$ correspond to the longitude and latitude of $p_i$, respectively, while $p_i.t_{in}$ and $p_i.t_{out}$ denote the timestamps of $p_1$ and $p_n$, respectively.

In order to achieve clustering of sub-stopovers, an Improved Density Peaks Clustering algorithm (IDPC) is proposed in this paper. Firstly, unlike traditional Density Peaks Clustering algorithms (DPC), a definition of the local dwell time density $\rho$ for $s_i$ is provided, as shown in Equations (3).

3 $$\begin{aligned} \rho _i= \sum _{i\ne j} \chi (d_{ij}-d_c)\Delta t_i \end{aligned}$$

where $d_{ij}$ represents the Euclidean distance between $s_{i}$ and $s_{j}$, while $d_c$ denotes the cutoff distance based on density peak clustering algorithm. $\chi (x)$ is a logical judgment function. If $x<0$, then $\chi (x)=1$; Otherwise, $\chi (x)=0$. $\Delta t_i$ represents the dwell time of the sub-stopover, calculated as $\Delta t_i=s_i.t_{out}-s_i.t_{in}$. It’s important to note that $\rho _i$ corresponds to the total residence time of other sub-stopovers that are less than or equal to $d_c$ from the sub-stopover $s_i$.

Secondly, the distance from the sub-stopover $s_i$ to the nearest sub-stopover $s_j$ with a higher local dwell time density is defined as $\gamma$, as shown in Equations (4).

4 $$\begin{aligned} \gamma _i= \min _{j:\rho _{j}>\rho _{i}}(d_{ij}) \end{aligned}$$

Thirdly, a decision graph is constructed with $\rho$ as the horizontal axis and $\gamma$ as the vertical axis. Utilizing thisdecision graph, sub-stopovers with higher values of $\rho$ and $\gamma$ are identified as cluster centers, while points with relatively low values of $\rho$ and relatively high values of $\gamma$ are labeled as noise points.

Finally, the remaining sub-stopovers are allocated using the following rule: each remaining sub-stopover is assigned to the cluster where its nearest neighbor and the sub-stopover with a higher density are located. Subsequently, all cluster centers are extracted as trajectory stopovers $S_i$. The extraction of stopovers is shown in Algorithm 1.

**Algorithm 2:**
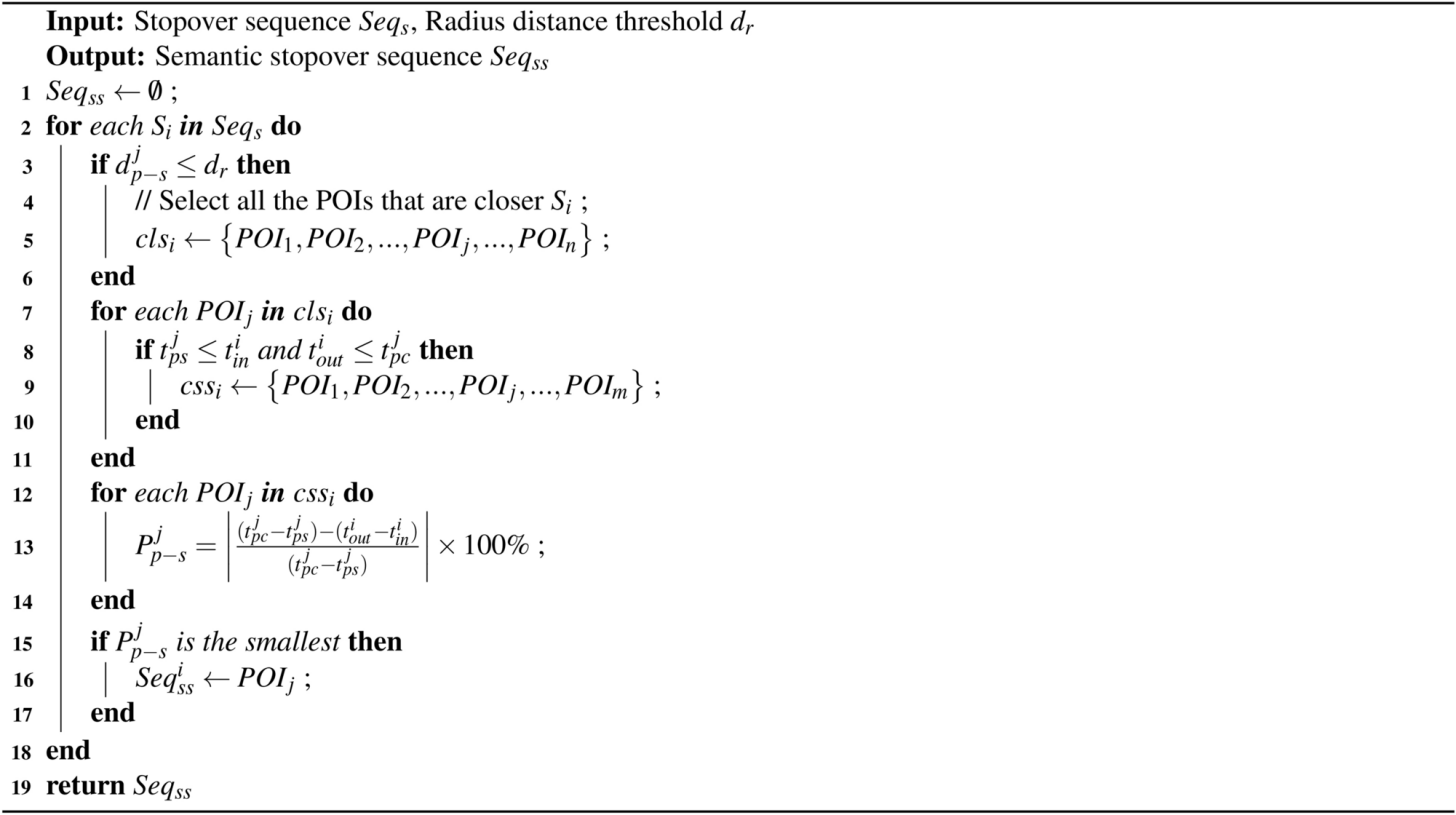
Location Semantic Mining Algorithm (LSMA)

### Mining semantics of stopovers

Due to the limited positioning accuracy of GPS devices and potential network delays in obtaining location data, there may exist discrepancies between the coordinates of sampling points and their actual locations. Moreover, sampling points might be missing, particularly in indoor spaces. Hence, it is unreasonable to directly determine the semantic category of a user’s stopovers based solely on their coordinates. Instead, it is necessary to match stopovers with nearby Points Of Interest (POIs). For example, Cheng *et al.*^40^ use the semantic categories of the nearest interest points to annotate stopovers. Since stopovers contain attributes such as coordinates, arrival time, and departure time, the Location Semantic Mining Algorithm (LSMA) proposed in this paper combines the categories of POIs near the stopover points with the attributes of these points

Firstly, a radius distance threshold $d_{r}$ is defined. Then, all POIs that are within a distance $d_{r}$ from the stopover $S_i$ are selected as candidate location sets $cls_{i}$, as shown in Equations (5).

5 $$\begin{aligned} cls_i= \left\{ POI_{1}, POI_{2},...,POI_{j},..., POI_{n} \right\} , d_{p-s}^{j} \le d_{r} \end{aligned}$$

where $d_{p-s}^{j}$ represents the distance from $POI_j$ to $S_i$, *n* is the number of POIs in $cls_i$.

Secondly, two quantitative features of the business hours of the POIs and the dwell time of the stopovers are selected as measurement indicators. From $cls_{i}$, the POIs that closely match the characteristics of the stopover are selected as candidate semantics sets $css_{i}$. Since users usually visit POIs during business hours, it is ensured that the arrival time of the stopover is later than the start time of the POI, and the departure time of the stopover is earlier than the closing time of the POI. As shown in Equations (6), POIs that do not meet these conditions in $cls_i$ are filtered out.

6 $$\begin{aligned} css_i= \left\{ POI_{1}, POI_{2},...,POI_{j},..., POI_{m} \right\} , t_{ps}^{j} \le t_{in}^{i}, t_{out}^{i} \le t_{pc}^{j}, m \le n \end{aligned}$$

where $t_{ps}^{j}$ is the start time of $POI_{j}$, $t_{pc}^{j}$ is the closing time of $POI_{j}$, *m* is the number of POIs in the filtered $css_i$.

Finally, the POI whose business hours most closely match the dwell time of the stopover $S_i$ from the $css_i$ is selected as the semantics of $S_i$. The proximity $P_{p-s}$ of the POI’s business hours and the dwell time of $S_i$ is calculated as shown in Equations (7). The process of mining semantics of stopovers is shown in Algorithm 2.

7 $$\begin{aligned} P_{p-s}^{j}= \left| \frac{(t_{pc}^{j}-t_{ps}^{j})-(t_{out}^{i}-t_{in}^{i})}{(t_{pc}^{j}-t_{ps}^{j})} \right| \times 100\% \end{aligned}$$

### Selection of candidate dummy positions

The selection of candidate dummy locations around the user’s stopovers is detailed in this section. The most ideal situation is that each stopover has $k-1$ dummy positions. In the trajectory data release scenario, the following privacy protection requirements need to be met.

**Fig. 9:**
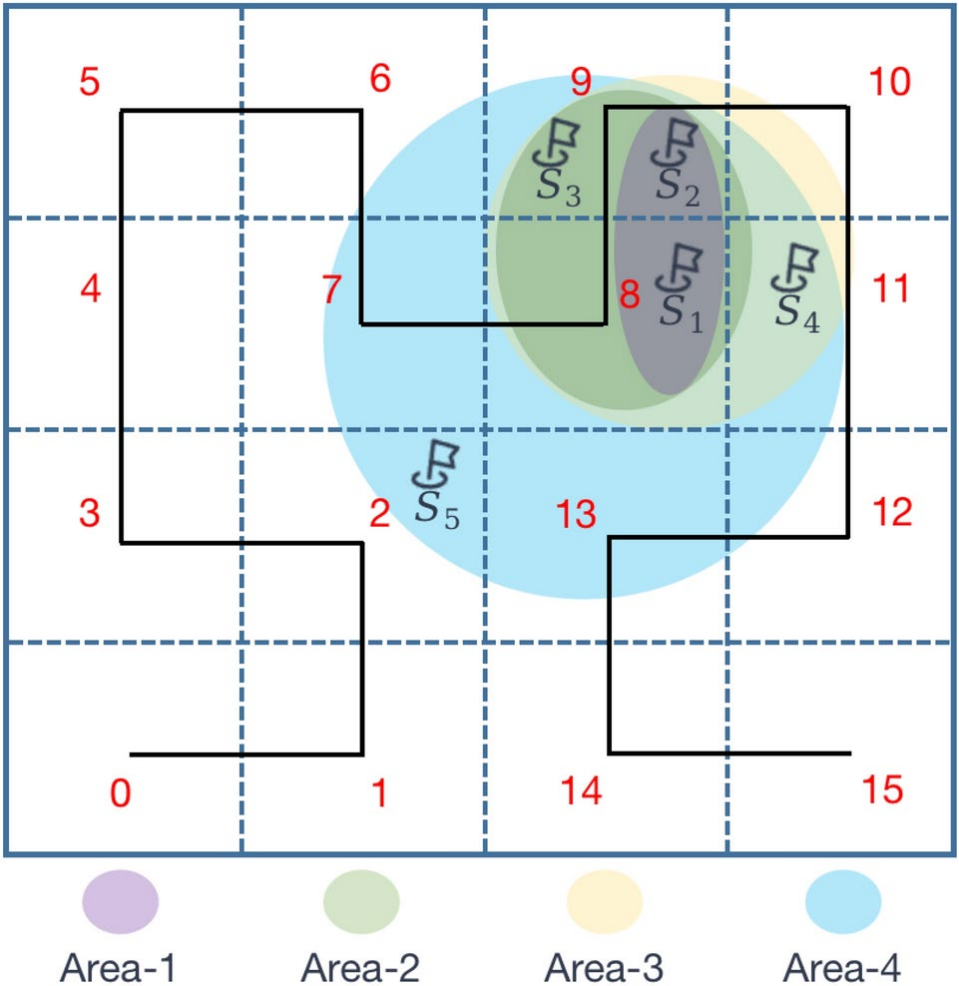
Anonymous areas selection based on Hilbert curve.

Firstly, the anonymity area formed by the anonymity set should ideally be as small as possible to minimize the impact on the availability of traffic trajectory data. To achieve this, the Hilbert curve^41^ is employed to select the dummy location closest to the real location, thereby forming an anonymity set. As shown in Figure 9, the Hilbert curve is a continuous fractal space-filling curve capable of linearizing multidimensional space into one-dimensional space. Specifically, the entire region is divided into $n \times n$ grids, and the Hilbert curve can linearly traverse each grid based on its spatial filling curve characteristics, passing through each grid only once. Each discrete grid is linearly sorted and encoded, providing a unique identifier for that grid. Through this encoding method, points with similar positions in two-dimensional space also have similar Hilbert values. For example, suppose the Hilbert values of the grids where the stopovers $S_1$, $S_2$, $S_3$, $S_4$ and $S_5$ are located are 8, 9, 9, 11 and 2. When $k=2$, $S_2$ will be selected as the dummy position in the anonymity set for $S_1$; When $k=3$, $S_2$ and$S_3$ will be chosen as the dummy positions in the anonymity set for $S_1$; When $k=4$, $S_2$, $S_3$ and $S_4$ will be chosen as the dummy positions in the anonymity set for $S_1$; When $k=5$, $S_2$, $S_3$, $S_4$ and $S_5$ will be chosen as the dummy positions in the anonymity set for $S_1$. According to this fake locations selection rule, the anonymous areas will be relatively concentrated, generating a smaller anonymous set, and the fake locations will also be relatively close to the real locations geographically.

Secondly, it is necessary to ensure that the selected dummy position is similar to the real position. Given the varying business hours and dwell times of different POIs, these two features are typically utilized to measure the similarity of activity patterns among POIs. Considering that the dwell time of POIs is normally distributed, a new similarity measurement method is proposed to solve this problem, utilizing six features to represent each POI: the position of the POI, the start time, the closing time, the expected dwell time, the variance of the dwell time, and the probability of user arrival in each hour interval at the POI. The feature vector of the POI can be represented as $\vec {poi}= \left[ loc,t_{ps},t_{pc},E_{dwe}, V_{dwe},P_{ari} \right]$, and cosine similarity is used to calculate the similarity of activity patterns between two types of POIs, as shown in Equations (8).

8 $$\begin{aligned} sim(poi_{i},poi_{j})=\frac{\vec {poi_{i}}\cdot \vec {poi_{j}} }{\left\| \vec {poi_{i}} \right\| \left\| \vec {poi_{j}} \right\| } \end{aligned}$$

where $poi_{i}$ and $poi_{j}$ represent different POIs. $sim(poi_{i},poi_{j})$ represents the similarity between two types of POIs, and the higher the value, the more similar the two types of POIs are. Therefore, a similarity threshold $\delta _{sim}$ is designed to select POIs with similar activity patterns to the user’s real location.

Thirdly, the number of semantic categories of POIs in anonymous sets should ideally be maximized. In the privacy protection process, when the activity patterns of POIs within each anonymity set closely resemble those of the real location, having a greater diversity of POI categories enhances the privacy protection effectiveness of the location data. The optimal scenario is to have $k-1$ different categories of POIs in each anonymity set.

The process of diversified semantic dummy location selecting is shown in Algorithm 3. The selection of dummy positions with different semantics is delineated in lines 11 to 23. The algorithm generates a multi-class nested POI set $Set_{mc}$ based on different semantic types of POIs in similar POIs sets $Set_{ap}$, wherein POIs with the same semantic category are grouped into the same subcategory set $Set_{sub}$. Subsequently, the algorithm iterates through $Set_{mc}$ and selects the $Set_{sub}$ with the highest number of POIs, randomly removing one POI from it until the final number of POIs in $Set_{mc}$ equals $k-1$, where *k* is the number of positions in the selected anonymity set.

**Algorithm 3:**
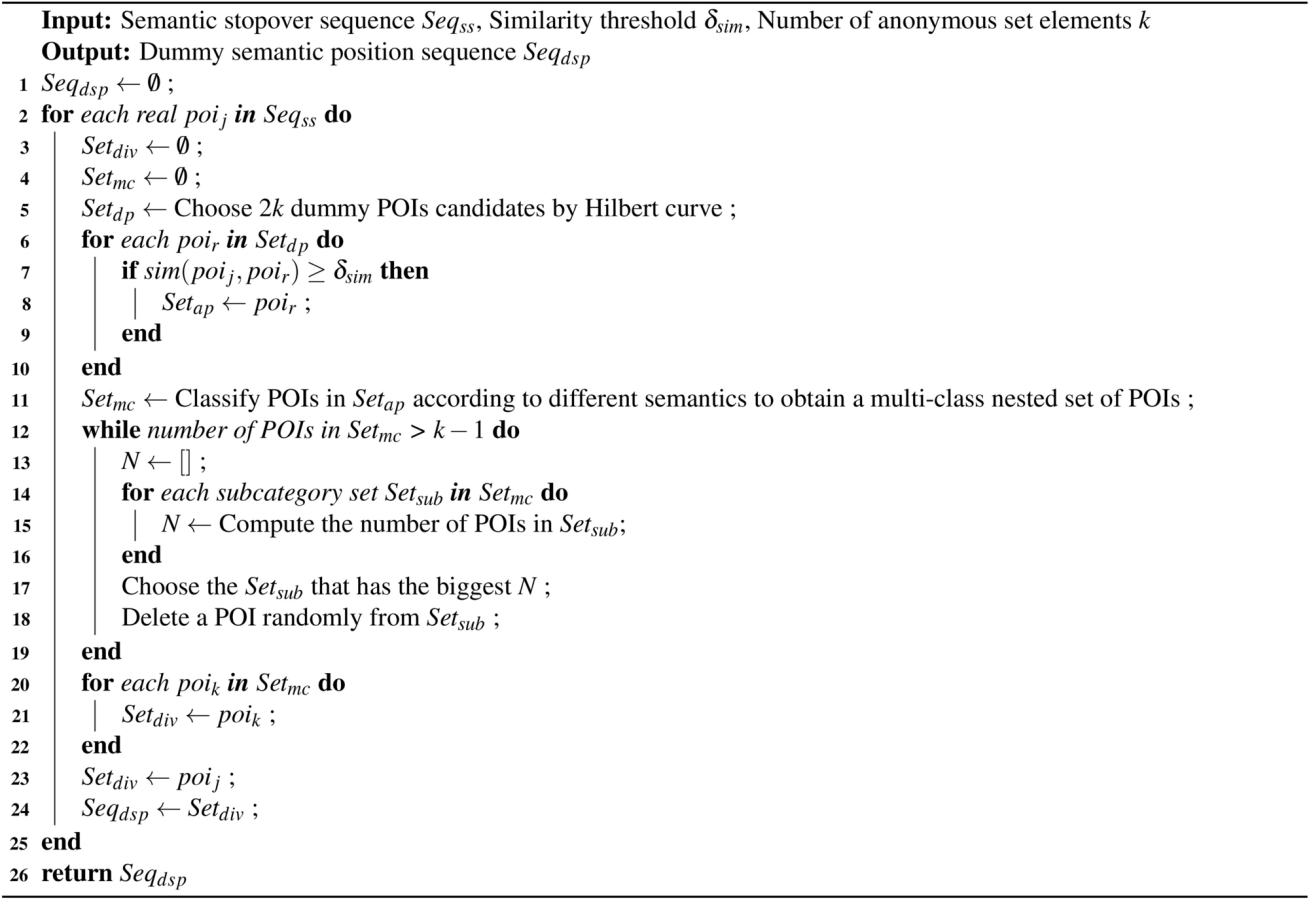
Diversified Semantic Dummy Location Selecting Algorithm (DSDLSA)

### Dummy trajectory synthesis

The detailed process of synthesizing dummy trajectories will be introduce in this section. While most studies typically focus on the rationality of adjacent anonymity sets during the synthesis of dummy trajectories, which involves comparing time reachability, directional similarity, and calculating entry and exit degrees, the LSPPM-SI model proposed in this paper aims to generate semantic locations that are challenging for attackers to recognize. In addition to the above three factors, LSPPM-SI also considers the spatial influence factors of interest points base on the multi semantic anonymous set. This consideration ensures that fake trajectories more effectively protect the user’s location both semantically and geographically.

Different POIs exhibit varying patterns of usage over time. The distribution of usage time for services provided by different POIs varies. For example, most restaurants experience higher user visits during lunch or dinner time, whereas other times witness fewer visits. Conversely, bars typically attract more customers at night and fewer during the day. To model this, each day is divided into 24-hour intervals. For POIs with different semantic subcategories, each hourly interval possesses different hourly access probabilities, which can be learned from real user traffic trajectory datasets. The spatial influence threshold $\delta _{I}$ is proposed in this paper, which uses the spatial influence of POIs to match the dummy positions of two adjacent anonymous sets. The access probability of a dummy location in a previous adjacent anonymous set accessing a dummy location in a subsequent adjacent anonymous set is shown in Equation (9).

9 $$\begin{aligned} Pr(p_{i+1}^{r}|p_{i}^{r})=\left\{ \begin{matrix} 0, & I_{i+1} \le \delta _{I} \\ I_{i+1}\cdot p_{ari}, & otherwise \end{matrix}\right. \end{aligned}$$

where $p_{i}^{r}$ represents the dummy position of the anonymous set of the previous stopover in a trajectory of user $U_i$, and $p_{i+1}^{r}$ represents the dummy position of the anonymous set of the next stopover. $I_{i+1}$ is the spatial influence of $POI_{i+1}$ on user $U_i$, $p_{ari}$ is the probability of$U_i$ arrivals in each hour interval of the $POI_{i+1}$.

In order to maximize the protection of user location privacy, the LSPPM-SI model compares the probability that the dummy positions in the anonymity set of stopover $S_i$ in each trajectory visit the dummy positions in the next stopover $S_{i+1}$ anonymity set to the probability of real stopover $S_i$ visiting the real stopover $S_{i+1}$. Dummy positions with relatively similar probabilities are matched into several dummy trajectories. Subsequently, the $k-1$ most similar access probabilities are selected from these dummy trajectories to generate anonymous set of dummy trajectories.

**Fig. 10:**
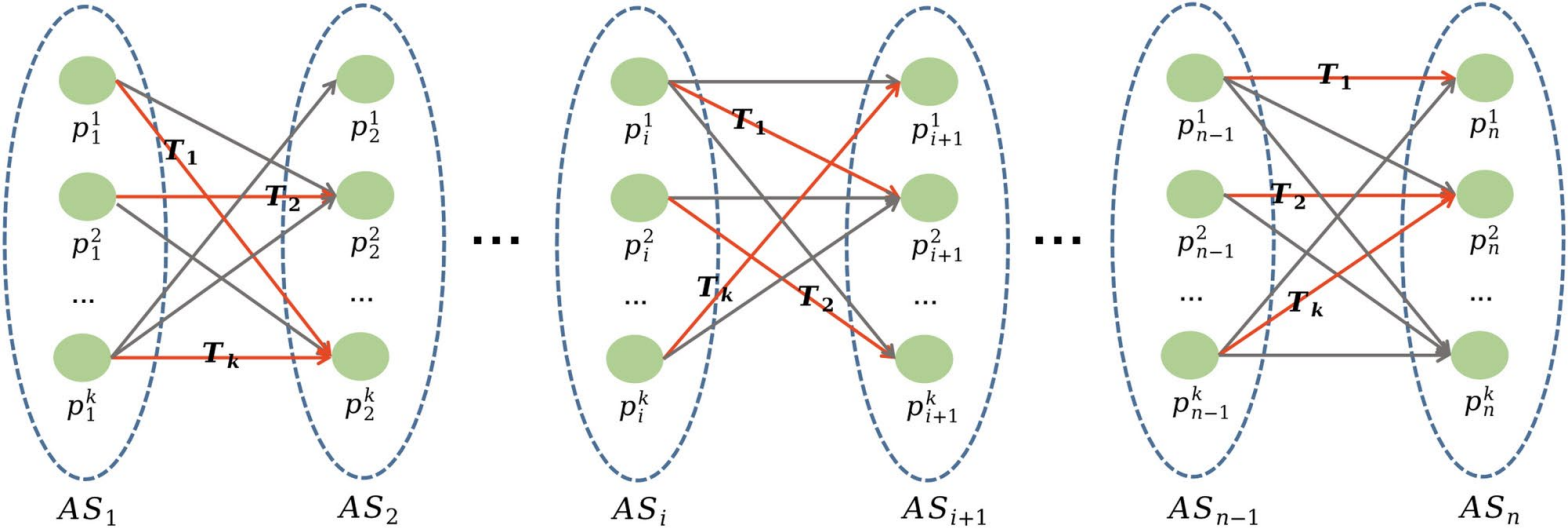
A bipartite graph generated by an anonymous sets of stopovers in a trajectory.

Let $AS_i=\left\{ p_{i}^{1}, p_{i}^{2},..., p_{i}^{k} \right\}$ represents the set of candidate dummy positions generated by stopover $S_i$ of the user’s real traffic trajectory, and $AS_{i+1}=\left\{ p_{i+1}^{1}, p_{i+1}^{2},..., p_{i+1}^{k} \right\}$ represent the set of candidate dummy positions generated by stopover $S_{i+1}$ of the user’s real trajectory. Both $AS_i$ and $AS_{i+1}$ include $k-1$ dummy location and one real position. As shown in Figures 10, $AS_{i}$ and $AS_{i+1}$ are used to generate directed weighted bipartite graphs. The positions of each anonymous set constitute the vertices of a bipartite graph, connected by directed edges. The weight of each edge represents the probability of the user accessing $p_{i+1}^{r}$ when located at the stopover $p_i^{r}$. Anonymous set of *n* stopovers can generate $n-1$ directed bipartite graphs. In each bipartite graph, the vertices on both sides match each other based on the access probability. According to Equation (9), if there exists an access probability, a directed edge connects the two vertices; otherwise, there isn’t. In order to make it more difficult to distinguish between the synthetic trajectory and the real trajectory, $k-1$ dummy position matching pairs with a more uniform probability distribution and closer to the access probability of real situation are selected in each bipartite graph. The $k-1$ dummy position matching pairs generated by each bipartite graph are then connected to the corresponding vertices to synthesize $k-1$ dummy trajectories. Subsequently, an anonymity set $T=\left\{ T_1, T_2,..., T_k \right\}$ is formed, comprising $k-1$ dummy trajectories and a real trajectory.

In order to select $k-1$ dummy position matching pairs with a more uniform probability distribution and closer to the access probability of real situation in each bipartite graph, this objective is approximated as the problem of finding the sum of squares of the minimum deviation for $k-1$ probabilities. Assuming $W_i$ is represented as the squared of minimum deviation between the access probability of the *i*-th dummy position matching pairs and the corresponding true situation, as shown in Equation (10).

10 $$\begin{aligned} W_i= (Pr(p_{i+1}^{r}|p_{i}^{r})-Pr(p_{i+1}^{real}|p_{i}^{real}))^2 \end{aligned}$$

where $Pr(p_{i+1}^{real}|p_{i}^{real})$ represents the access probability of the true situation. Then, the problem of generating an optimal anonymous set of trajectories can be transformed into an optimization problem as shown in Equation (11). The Kuhn Munkres algorithm^42^ is used to solve this optimization problem. The details of the dummy trajectory synthesis process are shown in Algorithm 4.

11 $$\begin{aligned} C=argmin( {\textstyle \sum _{i=1}^{k-1}W_{i}}) \end{aligned}$$

**Algorithm 4:**
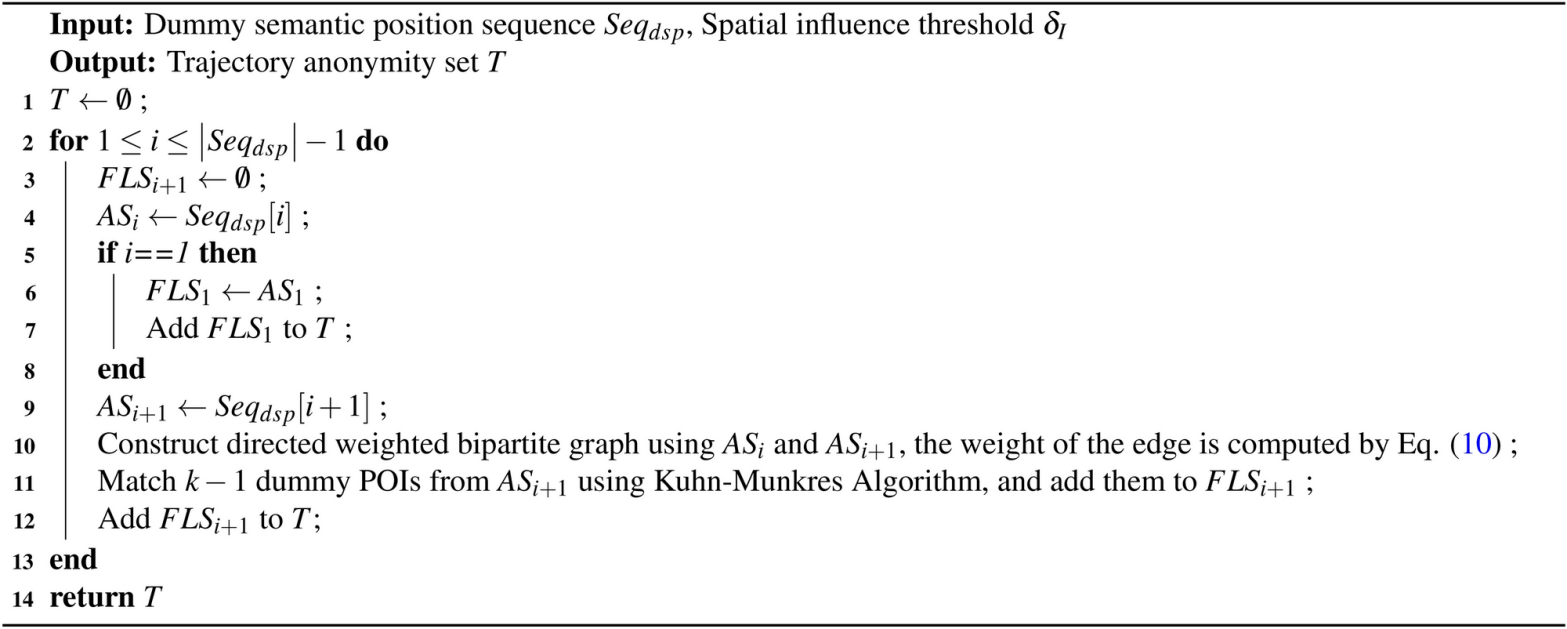
Dummy Trajectory Synthesis Algorithm (DTSA)

### Complexity analysis

To evaluate the feasibility of the proposed approach, we provide a theoretical analysis of its time and space complexity, focusing on the key components of the Location Semantic Privacy Protection Model based on Spatial Influence (LSPPM-SI).

Time Complexity: The time complexity of the proposed LSPPM-SI model can be analyzed based on its major processes: semantic dummy location selection, spatial influence analysis, and dummy trajectory synthesis. (1) Semantic Dummy Location Selection: In this step, dummy locations are selected from a pool of *n* candidate points based on their semantic similarity to the target location. To achieve sufficient diversity, each candidate location must be compared with the target location across *m* iterations. Therefore, the overall time complexity of this process is $O(n \times m)$, where *n* is the number of candidate locations and *m* is the number of iterations needed for diversity. (2) Spatial Influence Analysis: This step involves calculating the spatial influence between points of interest in each stopover’s anonymity set. Suppose the number of stopovers in a trajectory is *l*, and each stopover has an anonymity set of *k* points. For each stopover, we need to calculate the pairwise spatial influence within its anonymity set, which requires $O(k^2)$ operations. Since there are *l* stopovers in total, the overall time complexity for spatial influence analysis becomes $O(l \times k^2)$. (3) Dummy trajectory synthesis: Constructing an anonymous trajectory involves iterating over the sequence of *l* stopovers in a trajectory and creating anonymity sets of size *k*for each stopover. The complexity for creating all anonymity sets is $O(l \times k)$. When combined with the influence analysis, the total time complexity for trajectory anonymization becomes $O(l \times k^2)$. Therefore, the total time complexity of the proposed approach is approximately $O(n \times m + l \times k^2)$, which is manageable for moderate values of *n*, *m*, *k*, and *l*.

Space Complexity: The space complexity is determined by the storage requirements for candidate locations, semantic properties, and anonymity sets. (1) Candidate Locations Storage: The storage for *n* candidate locations requires *O*(*n*) space, where each candidate location is represented by its coordinates and semantic attributes. (2) Anonymity Sets: For each of the *l* stopovers in a trajectory, an anonymity set of size *k* is created. Therefore, the storage required for all stopovers is $O(l \times k)$. Additionally, storing the spatial influence factors between points requires $O(l \times k^2)$ space, since we need to store the influence for each pair of points in each anonymity set. (3) Auxiliary Data: The auxiliary data, such as semantic scores and spatial influence metrics, also require additional space, but this scales linearly with the number of locations or anonymity sets, contributing $O(k \times l)$. In total, the space complexity of the proposed method is $O(n + l \times k + l \times k^2)$, which indicates that the proposed method is feasible for typical datasets where the number of candidate locations and stopovers are within a reasonable range.

The theoretical analysis suggests that the proposed LSPPM-SI model is computationally feasible for practical applications, with both time and space complexities scaling reasonably with the number of candidate points, stopovers, and anonymity sets. The linear and quadratic scaling ensures that the method can be efficiently applied to trajectory datasets without requiring excessive computational resources.

## Experiments

### Experimental environment

In this section, the method proposed in this paper is simulated and analyzed. The simulation platform utilized is PyCharm, employing Python 3.9. The code runs on the macOS operating system with a hardware environment on macOS v13.0 with a configuration consisting of a 10-core CPU of M1 Max and 16 GB memory. The experimental dataset utilized is based on real dataset Geolife. This dataset comprises 17,621 GPS trajectories from 182 users spanning over a period of more than 5 years (from April, 2007 to August, 2012). The points of interest in this dataset are divided into 12 lager categories, 125 medium categories, and 1216 subcategories, with a total of 562364 points of interest. The data records are stored in PLT format, containing information such as latitude, longitude, date, time, etc., as shown in Table 3.

**Table 3:** The data storage format of the Geolife dataset.

Number	Field Name	Data type	Unit	Example
1	Latitude	Float	Degree	39.984702
2	Longitude	Float	Degree	116.318417
3	Altitude	Float	Feet	492
4	Date	Char	Null	2008-10-23
5	Time	Char	Null	02:53:04

### Evaluation Indicators

Three evaluation indicators are used in this paper to measure the degree of privacy protection and data availability of the proposed model. Access entropy is used to measure the performance of location privacy protection; Information loss is used to measure the utility of traffic trajectory data. Semantic protection degree is used to measure the performance of semantic protection. The specific definitions of these indicators are as follows:

(1) Access entropy

Access entropy is used to measure the degree of anonymity, reflecting the uncertainty in determining the user’s actual current position from a set of anonymous fake locations. When all *k* dummy POIs have the same access probability, the access entropy reaches its maximum. The access entropy *AE* is calculated by Equation (12).

12 $$\begin{aligned} AE=- {\textstyle \sum _{i=1}^{k}Pr(p_i | u_i)\cdot \log _{2}{Pr(p_i | u_i)} } \end{aligned}$$

where $Pr(p_i | u_i)$ is the probability of user $u_i$ accessing $p_i$.

(2) Information loss

Information loss refers to the loss of original traffic trajectory information caused by anonymity of traffic trajectories, which demonstrates the data availability after trajectory publication. For a published trajectory, each real stopover is generalized to an area containing at least $k-1$ dummy positions. Information loss is measured by the sum of the areas of anonymous regions, calculated by Equation (13).

13 $$\begin{aligned} IL= {\textstyle \sum _{i=1}^{n}Area(x_i, y_i, r_i)} \end{aligned}$$

where $x_i$ and $y_i$ represent the coordinates of the real stopover, and $r_i$ represents the radius of the anonymous area where the stopover is located.

(3) Semantic protection degree

The semantic privacy protection effect improves with an increase in the number of categories of POIs in each anonymity set. In this paper, the average number of subcategories is utilized as a measure of semantic privacy protection. Given a traffic trajectory *Traj*, the anonymous set of trajectories is denoted as *T*. Let $|S_i|$ represent the number of stopovers extracted from *Traj*, and $AS_{S_i}$ denote the anonymity set of stopover $S_i$. The number of POI categories in $AS_{S_i}$ is denoted as $C_{S_i}$. The semantic privacy protection degree *SD* can be calculated by Equation (14).

14 $$\begin{aligned} SD= \frac{ {\textstyle \sum _{i=1}^{|S_{i}|}C_{S_i}} }{|S_{i}|} \end{aligned}$$

### Performance analysis

Three traffic trajectory privacy protection methods, namely the Random method, the LPP method^43^, and the STP method^32^, are compared with the LSPPM-SI method proposed in this paper. Random method randomly selects $k-1$ dummy positions to form an anonymity set. The LPP method aims to provide higher transfer entropy while keeping the grid entropy measurement close to optimal. The STP method generates $k-1$ dummy trajectories in sensitive areas based on the user’s motion mode, road network topology, and the proportion weight of road categories in the sensitive area. In the LSPPM-SI proposed in this paper, each anonymous set is designed to contain as many interest point categories as possible, and each dummy position in adjacent anonymous sets satisfies the spatial influence condition. The final performance is determined based on the average of 100 experiments. This comparison will shed light on the effectiveness of the LSPPM-SI method compared to existing approaches in terms of traffic trajectory privacy protection.

**Fig. 11:**
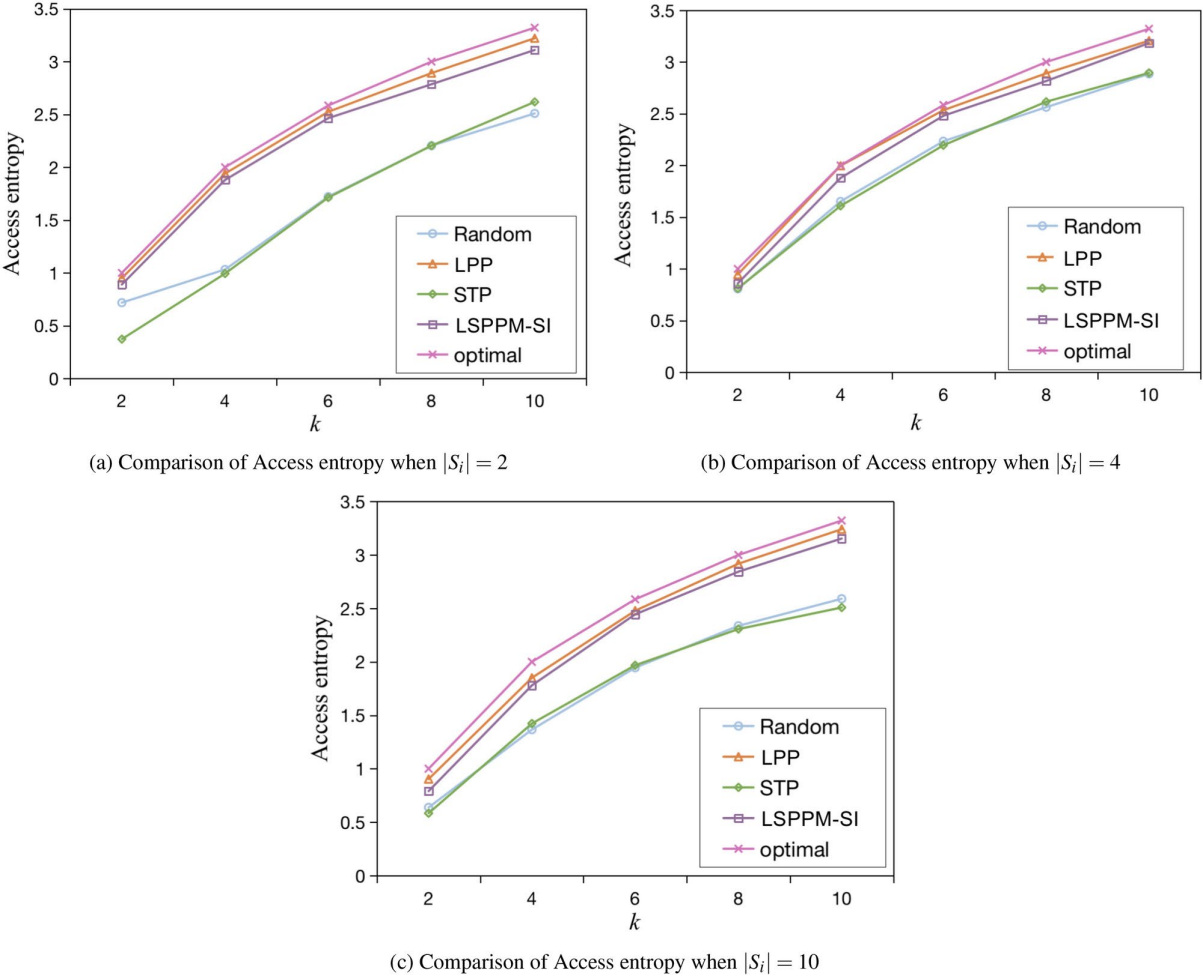
Comparison of Access entropy for different algorithms.

(1) Access entropy analysis

As shown in Figures 11 (a) - (c), the performance of different algorithms in terms of access entropy is compared when the number of stopovers $|s_i|$ are 2, 4, and 10, respectively. When *k* access probabilities are equal, the access entropy $AE=\log _{2}{k}$ reaches its maximum value. This maximum value represents the optimal achievable access entropy for all algorithms. In the Random method, all dummy positions of the anonymous set are randomly selected, resulting in significant differences among these *k* access probabilities. Consequently, the access entropy of the Random method is lower compared to the other three algorithms. This implies that attackers can easily identify most dummy positions within a continuous anonymity set. The STP method focuses on protecting the semantic privacy of users but disregards the spatial influence of interest points. Consequently, many dummy location access probabilities differ from the actual user access probabilities. Therefore, the access entropy of the STP method is nearly equivalent to that of the Random method. In contrast, the access entropy of the LPP method and the LSPPM-SI method closely approaches the optimal access entropy. Attackers cannot exploit the access entropy to identify dummy positions effectively. The access entropy of the LPP method is slightly higher than that of the LSPPM-SI method, by approximately 3%. This difference arises because the LPP method selects the dummy position with the maximum grid entropy and transfer entropy.

**Fig. 12:**
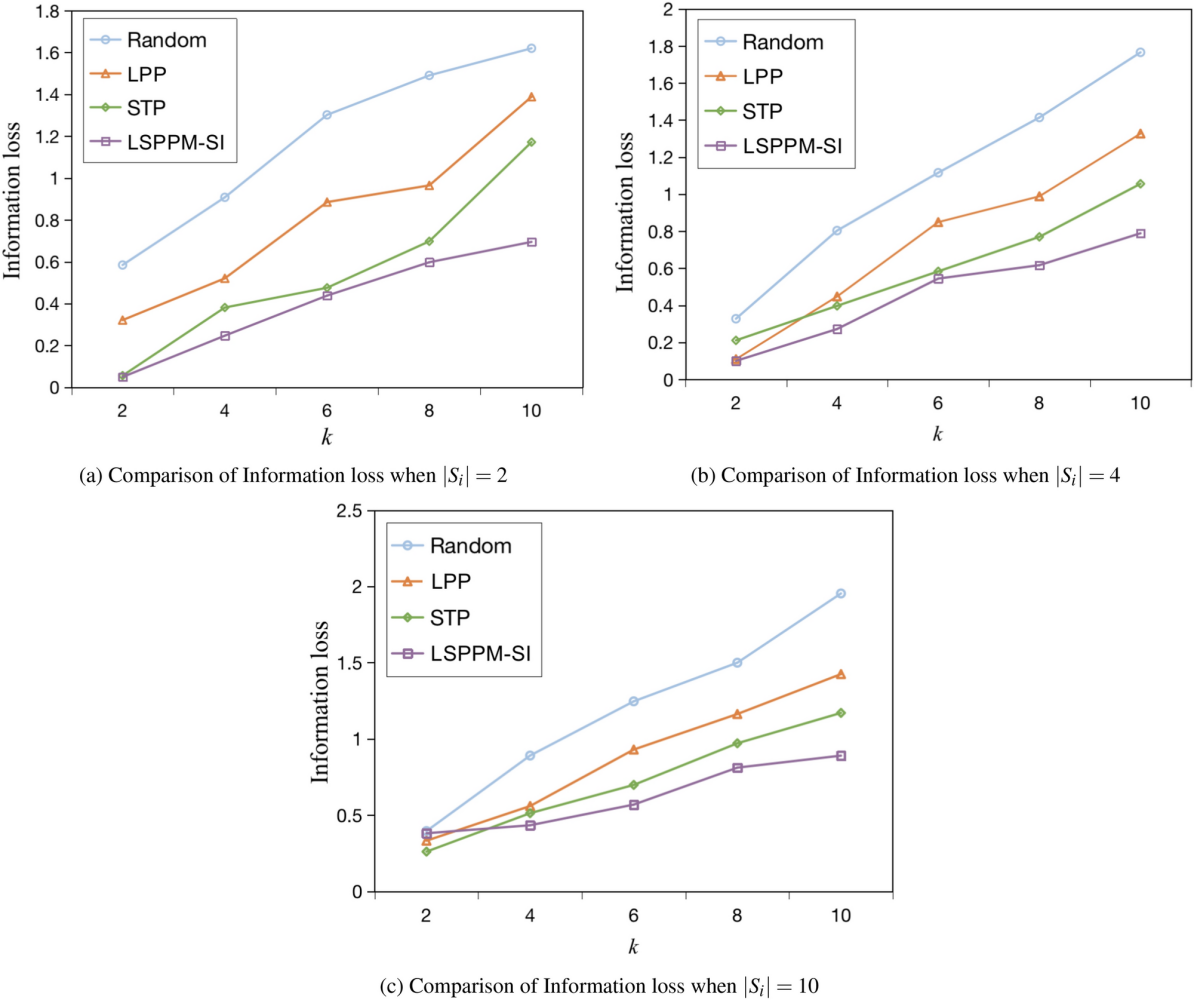
Comparison of Information loss for different algorithms.

(2) Information loss analysis

As shown in Figures 12 (a) - (c), the performance of different algorithms in terms of information loss is compared when the number of stopovers $|s_i|$ is 2, 4, and 10, respectively. Since each semantic position occupies a certain area, constructing *k*-anonymity sets requires a larger area. Generally, the larger the value of *k*, the larger the area of the anonymous region. From the graph, it is evident that both the STP method and the LSPPM-SI method exhibit lower information loss compared to the Random method and the LPP method. Specifically, the information loss of the STP method is 23% lower than that of the LPP method. The Random and LPP methods do not consider the size of anonymous regions when generating *k*-anonymous sets, resulting in unevenly distributed dummy positions and consequently large anonymous regions. In contrast, the STP method utilizes the Hausdorff distance spatial similarity algorithm to select positions with similar spatiotemporal distances, thereby reducing information loss compared to the Random and LPP methods. The LSPPM-SI method proposed in this paper demonstrates the best information loss performance, being 14% lower than the STP method. This improvement can be attributed to the use of the Hilbert curve in the selection process of initial candidate dummy positions, which ensures that the selected dummy positions are in close proximity to each other, thereby reducing the area of anonymous regions and significantly reducing information loss.

(3) Semantic protection degree analysis

**Fig. 13:**
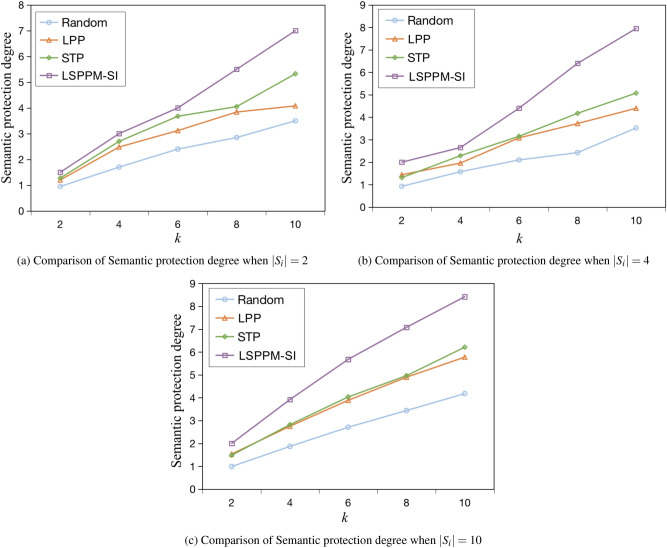
Comparison of Semantic protection degree for different algorithms.

As shown in Figures 13 (a) - (c), the performance of different algorithms in terms of semantic protection performance is compared when the number of stopovers $|s_i|$ is 2, 4, and 10, respectively. The semantic protection performance improves with an increase in the number of POI semantic categories in an anonymity set. In theory, the number of semantic categories in an anonymity set must be greater than or equal to 1 and not greater than *k*. When the number of semantic categories equals *k*, the optimal semantic privacy protection performance is achieved. From the graph, it is observed that the number of semantic categories for all four algorithms increases with the increase of *k*. The Random method selects dummy positions randomly, resulting in poor semantic protection performance, indicating that attackers can easily recognize the user’s positional semantics. The STP method generates a number of semantic categories that is 9% higher than that of the LPP method, but their semantic protection performance remains low. The number of semantic categories generated by the LSPPM-SI method proposed in this paper is 46.5% higher than that of the STP method. This indicates that the proposed semantic diversity position selection algorithm effectively generates more semantic categories. As the value of *k* increases, the semantic protection values of the four algorithms grow slower and slower. This is because there is a maximum limit on the number of semantic categories in the POIs dataset. Once *k* exceeds the upper limit of this value, there are duplicate semantic categories in the anonymity set, resulting in a smoother curve.

To further illustrate the characteristics of the original and anonymized trajectories, Table 4 provides an example of an original trajectory and its corresponding anonymized trajectories for a user. It includes the extracted stopovers and the generated anonymity sets ($k=3$) for each stopover, showcasing the process of generating dummy positions andanonymized trajectorys to ensure user privacy.

**Table 4:** Example of one original trajectory and its corresponding anonymous trajectory of a user for illustration.

Origin trajectory	[39.900302,116.385598]->...->[39.899347,116.387303]->...->[39.901005, 116.377466]
Extraction of stopovers	[39.900109,116.385788]->[39.899403,116.38604]->[39.899352,116.387422]->[39.89944,116.387874]
S1 anonymity set :	S1: [39.900109,116.385788], S1’: [39.90015,116.385701],
([39.900109,116.385788])	S1”: [39.900109,116.385788]
S2 anonymity set :	S2: [39.899403,116.38604], S2’: [39.899646,116.385897],
([39.899403,116.38604])	S2”: [39.899536,116.38658]
S3 anonymity set :	S3: [39.899352,116.387422], S3’: [39.899429,116.387113],
([39.899352,116.387422])	S3”: [39.89944,116.387977]
S4 anonymity set :	S4: [39.89944,116.387874], S4’: [39.899432,116.387834],
([39.89944,116.387874])	S4”: [39.899449,116.376099]
	T: S1->S2->S3->S4;
Trajectory anonymity set	T’: S1’->S2”->S3’->S4”;
	T”: S1”->S2’->S3”->S4’;

## Conclusions

It is a challenging issue that resisting location semantic attacks and improving data utility in trajectory data publishing based on LBS. To solve this problem, a Location Semantic Privacy Protection Model based on Spatial Influence (LSPPM-SI) is proposed in this paper. Firstly, the stopovers in trajectory data are extracted, and a location semantic mining algorithm is proposed to obtain the semantic information of each stopover. Secondly, in order to resist location semantic attacks, a diversified semantic location selection algorithm is proposed. The Hilbert curve is utilized to minimize the area of anonymous regions, thus enhancing the availability of traffic trajectory data. Thirdly, the spatial influence of interest points is defined and used to validate the rationality of dummy trajectories within the anonymous set of trajectories. This measure prevents dummy trajectories from being recognized by potential attackers. Finally, the synthesis of dummy trajectories is framed as a matching problem for directed bipartite graphs. The optimal *k*-anonymous trajectory set is then determined using the Kuhn Munkres algorithm. Comparative experimental results against traditional models demonstrate the superiority of LSPPM-SI in terms of traffic trajectory data availability and semantic protection performance. This comprehensive approach ensures both robust privacy protection against location semantic attacks and enhanced data utility for trajectory data publishing in LBS scenarios.

The LSPPM-SI proposed in this paper has demonstrated promising performance in safeguarding user location semantics. To enhance its practical utility, future endeavors will focus on integrating this location semantic privacy protection model with interest point recommendation applications. Additionally, we plan to explore how time information can be integrated into the model to further optimize wayfinding and route calculations. This will involve investigating the relationship between temporal characteristics of stopovers and dynamic path calculations, potentially enhancing the efficiency of the wayfinding algorithm.

The envisioned direction involves transitioning the model from its current application in trajectory publishing to real-time location semantic protection within the LBS service process. By integrating LSPPM-SI with interest point recommendation systems, and leveraging time information to improve routing, the aim is to uphold user location semantics while mitigating the loss of recommendation accuracy and enhancing computational efficiency. Additionally, future work will involve conducting experiments to empirically evaluate the time and space efficiency of the proposed approach, as well as testing it on additional datasets to further validate its effectiveness and scalability.

## Data Availability

The datasets used and/or analysed during the current study available from the corresponding author on reasonable request.
